# c-Met as a new marker of cellular senescence

**DOI:** 10.18632/aging.101961

**Published:** 2019-05-13

**Authors:** Maria Boichuck, Jonathan Zorea, Moshe Elkabets, Marina Wolfson, Vadim E. Fraifeld

**Affiliations:** 1 The Shraga Segal Department of Microbiology, Immunology and Genetics, Center for Multidisciplinary Research on Aging, Ben-Gurion University of the Negev, Beer Sheva 8410501, Israel

**Keywords:** cellular senescence, c-Met, Akt, Stat3, human dermal and pulmonary fibroblasts

## Abstract

Here, we reported for the first time an increased expression of c-Met protein in primary cultures of human dermal and pulmonary fibroblasts of late passages. This suggests that c-Met could serve as an early marker of cellular senescence (CS). The levels of c-Met-related signaling proteins phospho-Akt and Stat3 were also increased in (pre)senescent fibroblasts. Considering the anti-apoptotic activity of Akt and the involvement of Stat3 in mediating the effects of proinflammatory cytokines, the findings of this study indicate that c-Met could contribute through its downstream targets or partners to at least two major phenotypical features of CS – resistance to apoptosis and senescence-associated secretory phenotype.

## Introduction

In the last years, the phenomenon of cellular senescence (CS) has been becoming a hot topic in biomedical research. Apart from being involved in tissue repair upon wound healing [1,2] and acute fibrosis [3,4], cell reprogramming [5], and tissue remodeling during embryonic development [6–10], CS has attracted lots of attention, first and foremost, due to its strong links with aging and age-related diseases [11,12]. Indeed, the growing body of evidence indicates that accumulation of senescent cells could be an important player in mechanisms of aging and late-onset pathologies [13–16]. Senescent cells are characterized by a stable cell growth arrest, enlarged, flattened shape of heterogenous morphology, resistance to apoptosis, secretion of a plethora of proinflammatory and ECM-modifying compounds, and expression of several molecular markers (e.g., p16^INK4a^, p21^Cip1/Waf1^, SA-β-gal, etc.) [17]. Yet, none of the existing molecular markers is exclusively indicative of CS, and there are ongoing attempts for reliable identification of senescent cells [18,19].

While searching for microRNAs with a potential involvement in CS, we observed a profound down-regulation of miR-199a-3p and miR-34a in pre-senescent human skin fibroblasts [20]. Among their experimentally validated targets is the hepatocyte growth factor receptor (MET), a well-known proto-oncogene encoding for the c-Met protein with tyrosine kinase activity [21]. Then, it would be reasonable to suggest that a decreased expression of miR-199a-3p and miR-34a in (pre)senescent cells could result in overexpression of c-Met. Indeed, elevated levels of c-Met were reported for a variety of tumors in which the members of miR-34 and miR-199 families are often silenced (reviewed by [22]). Not surprisingly, c-Met has extensively been studied in cancer research [21]. The studies on c-Met with regard to CS are fully absent.

The c-Met receptor can interact with a number of signaling proteins. As a result, this interaction may lead to induction of various signaling pathways (PI3K/AKT, JAK/STAT, etc.), thus explaining a wide range of c-Met biological activities including cell survival, migration and adhesion [21,23,24]. Of note, all of these activities could be relevant to CS.

This study was undertaken to evaluate whether the expression of c-Met is altered in the course of replicative CS, and if so, whether its levels could serve as a new marker of senescent cells. Also, to get insight into possible role of c-Met in CS, we determined the expression of its downstream targets Akt and Stat3.

## RESULTS

### Expression of c-Met protein in primary cultures of human fibroblasts

The patterns of cell growth and CS of primary cultures of human fibroblasts were similar to those described by us previously [25,26]. Briefly, fibroblasts (both dermal and pulmonary) of early passages (P.12-20) displayed a typical spindle-like shape, did not express SA-β-gal, and doubled their population each 48-72 h. In contrast, the fibroblast cultures of late passages (around P.35-40) had heterogenous morphotypes, with large cells of irregular shape, that were stained with CS marker SA-β-gal, and dramatically slowed down their growth (PDT 3-4 weeks) or ceased to divide. Altogether, this allows to characterize the cell cultures of late passages as pre- or senescent cultures.

**Figure 1:**
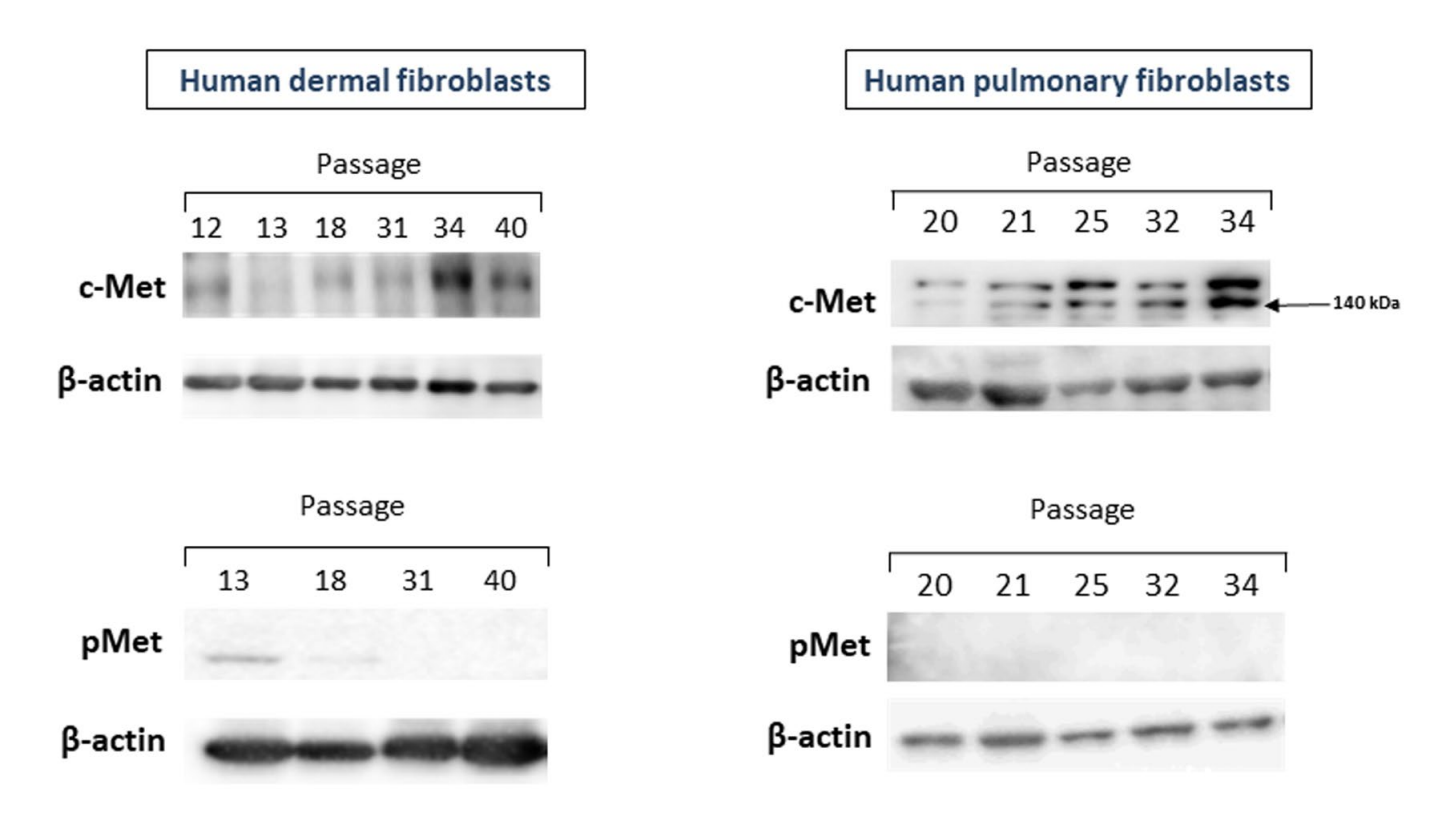
The levels of c-Met and pMet proteins in primary cultures of human dermal (left panel) and pulmonary fibroblasts (right panel) of various passages. Immunoblots represent one of the 4 independent experiments.

We first measured the levels of c-Met protein as well as its phosphorylated form (pMet) in primary cultures of human fibroblasts of various passages and different origin (dermal and pulmonary fibroblasts). As seen in Figure 1, both dermal (left panel) and pulmonary (right panel) fibroblasts displayed similar patterns of changes in c-Met protein levels during the course of replicative CS.

The c-Met protein was hardly detectable in cell cultures of dermal fibroblasts of early passages (P.12-18), and this low level remained up to P.31. A marked increase in the c-Met level was observed in cell cultures of late passages (P.34-40), when the cell growth dramatically slowed down. In pulmonary fibroblast cultures, the level of c-Met started to gradually increase from P.25, with the peak in P.34. As in case of dermal fibroblasts, the c-Met protein levels coincided well with population doubling time (PDT), so that the longer PDT, the higher c-Met level. It should be noted that a clear elevation of c-Met levels was observed before reaching the full growth arrest. Yet, PDT of senescing cultures of the examined late passages was quite long (3-4 weeks) and most cells were stained with the CS marker SA-β-gal. In contrast to c-Met, its phosphorylated form pMet was either undetectable in cell cultures of both early and late passages (pulmonary fibroblasts) or was of a very low level in only early passages but not late ones (dermal fibroblasts) (Figure 1, left-lower panel).

### Expression of Akt in primary cultures of human fibroblasts

**Figure 2:**
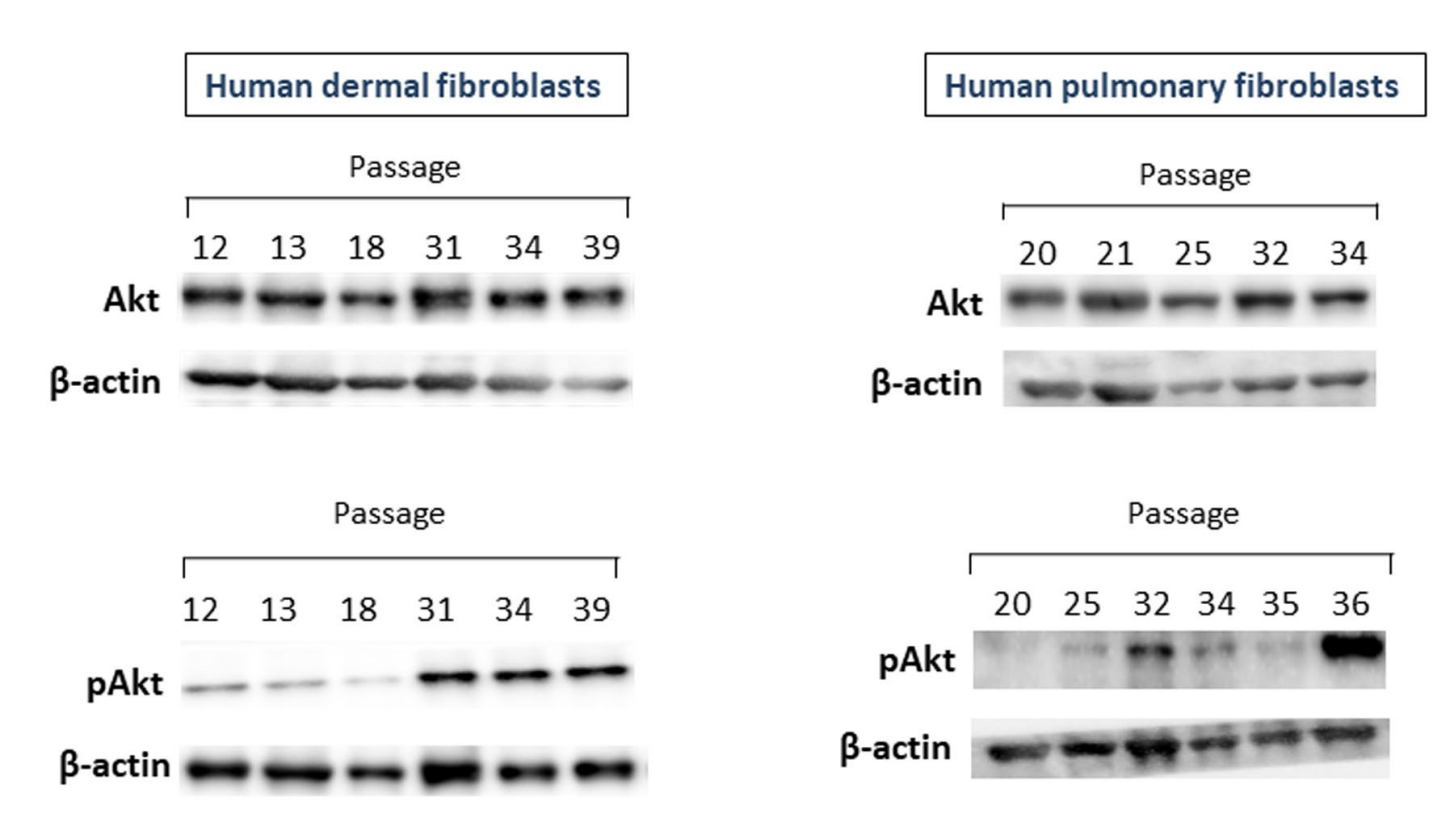
The levels of Akt1/2/3 and pAkt proteins in primary cultures of human dermal (left panel) and pulmonary fibroblasts (right panel) of various passages. Immunoblots represent one of the 3 independent experiments.

One of the c-Met downstream pathways which could be related to CS is a PI3K/AKT pathway. With this in mind, we further examine whether Akt1/2/3 and its phosphorylated form pAkt undergo changes during replicative CS. As seen in Figure 2 (upper panel), no significant differences in the Akt protein level between fibroblast cultures of late and early passages were observed, while pAkt unexpectedly increased in fibroblast cultures of late passages (lower panel). This patterns of CS-related changes in Akt and pAkt were common for both dermal and pulmonary fibroblasts. As in the case of c-Met, the levels of pAkt tended to inversely correlate with the number of passages, i.e., the higher the passage number (and, accordingly, the lower cell growth rate), the higher the level of pAkt.

### Expression of Stat3 in primary cultures of human fibroblasts

**Figure 3:**
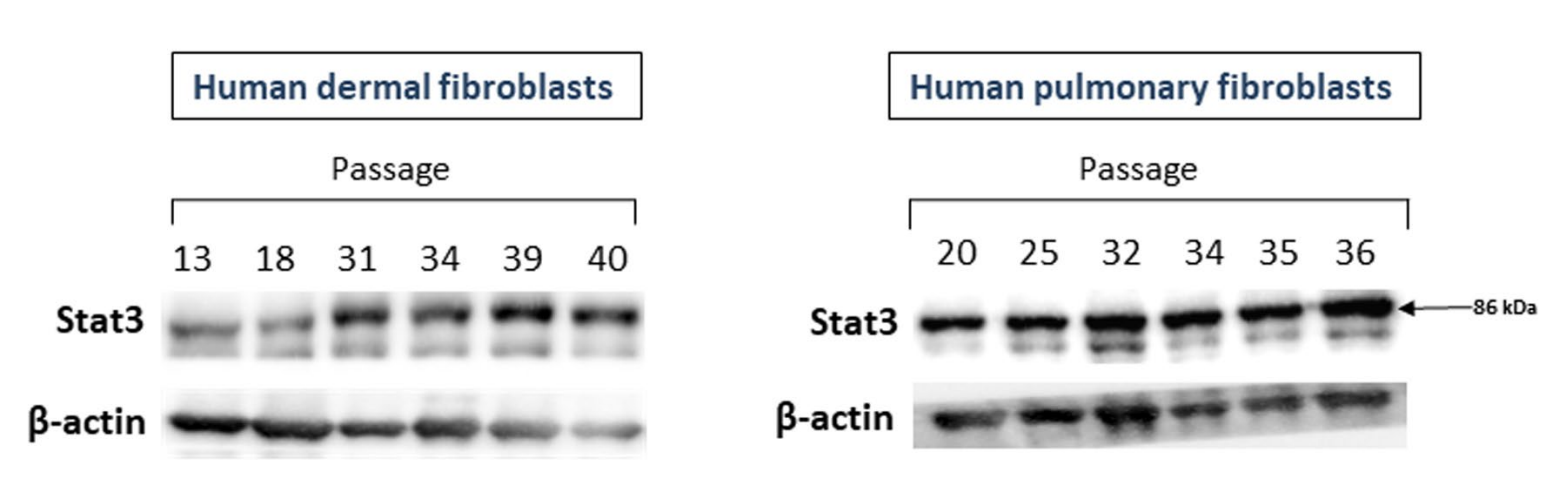
The levels of Stat3 proteins in primary cultures of human dermal (left panel) and pulmonary fibroblasts (right panel) of various passages. Immunoblots represent one of the 3 independent experiments.

The c-Met target Stat3 could also be directly related to CS. Therefore, we examined the levels of Stat3 protein in fibroblast cultures of various passages. As seen in Figure 3, the expression of Stat3 protein increased in fibroblast cultures of late passages. This was particularly noted for dermal fibroblasts (left panel) and, to a lesser degree, for pulmonary fibroblasts (right panel).

## DISCUSSION

In this study, we examined the expression of c-Met protein in fibroblast cultures of various passages, from early to late ones. The rationale was based on our recent finding, indicating that downregulation of several miRNAs could govern CS through overexpression of their targets, c-Met included (see Introduction). In line with this suggestion, we found for the first time an increased expression of c-Met protein in primary cultures of dermal and pulmonary fibroblasts during the course of replicative CS (Figure 1). Notably, in fibroblasts of early passages, the c-Met level was very low or undetectable, which is consistent with previously reported data [27,28]. The high c-Met protein levels were clearly evident in presenescent fibroblast cultures. This suggests that c-Met could serve as an early marker of CS. Two lines of observations indirectly support this notion. First, it was shown that both *in vitro* and *in vivo*, myofibroblasts (activated fibroblasts) express c-Met [29]. On the other hand, it was also found that senescent fibroblasts express the myofibroblast-specific marker α-SMA, thus connecting CS with myofibroblasts [25,30]. Second, transfection of normal dermal fibroblasts with the IL-1α gene whose overexpression is an initiation event in SASP development [31], resulted in high expression of c-Met mRNA [27]. Of note, the c-MET protein is expressed in a variety of cell types including epithelial, muscle, neuronal cells, etc [29,32]. Whether senescent cells other than dermal and pulmonary fibroblasts also express high levels of c-Met needs to be clarified.

As mentioned above, elevated levels of c-Met could be related to a decreased expression of miR-199a-3p and miR-34a. Downregulation of these microRNAs was consistently observed in cancer cells [22] and was also shown by us in (pre)senescent fibroblast cultures [20]. Another possibility for c-Met accumulation in both cancer and senescent cells could include its inadequate ubiquitination [33]. Whatever the case, it would be attractive to speculate that the proto-oncogene c-Met might induce CS by the well-known mechanism [34] of oncogene-induced cellular senescence.

Further complicating the cancer-CS relationships is a recently discovered HGF-associated mechanism by which the cancer cells may induce CS in normal cells that, in turn, form a cancer-promoting microenvironment [35,36]. However, whether c-Met mediates the CS-promoting effects of HGF has not yet been established.

In contrast to c-Met, we did not find any detectable passage-dependent elevation in its activated form pMet (Figure 1). It should however be noted that we determined only two (Tyr1234 and Tyr1235) of 14 sites of phosphorylation in c-Met that have thus far been identified [21]. Although phosphorylation of c-Met at Tyr1234 and Tyr1235 is believed to be a critical event in the c-Met kinase activity [37], the role of other phosphorylation sites cannot be excluded. Besides, activation of c-Met could be related to the production of its ligand—hepatocyte growth factor (HGF) [38,39]. Yet, the studies on HGF in CS are scarce and controversial. In early study by Miyazaki et al. (1998), an increased production of HGF by human embryonic lung fibroblasts of late passages vs. early passages was reported [40]. The authors also showed that the human skin fibroblasts derived from the old donors (80+ years) produced more HGF than the cells from the young and middle-aged donors. However, these findings were not confirmed by recent *in vitro* and *in vivo* studies on human skin fibroblasts. Qin et al. (2017) demonstrated that HGF mRNA was similarly expressed in the fibroblasts isolated from the skin of young (mean age 27±1 years) and aged (mean age 83±1.4 years) donors, as well as in fibroblast cultures of early passages and late passages (PDT of 45 days) [41]. Whatever the case, a possible role of HGF/c-Met axis in CS warrants further investigation.

c-Met receptor is a transmembrane protein which directly or indirectly interacts with numerous partners [37,https://thebiogrid.org/]. Among them, at least several could be relevant to CS. In this study, we focused on Akt and Stat3 proteins that are well known for their anti-apoptotic activity and mediating the effects of proinflammatory cytokines, respectively.

Unexpectedly, the opposite of c-Met picture was observed for Akt which is activated via interaction of c-Met with PI3K, directly or by forming a protein complex with GAB1 [37]. While there were no significant differences in the Akt protein level between fibroblast cultures of late and early passages, the levels of its active form pAkt was markedly increased in (pre)senescent fibroblasts (Figure 2). It should be noted that apart from c-Met, several other signaling pathways (e.g., EGF/EGFR, INS/IGF-1) could also activate the Akt protein [43]. Of note, among the major downstream effectors of Akt is a serine/threonine kinase mTOR [44], known to be strongly associated with CS and aging (for recent review see [45]). The levels of another c-Met target, Stat3 protein, a member of signal transducers and activators of transcription (JAK/STAT) pathway, also increased in fibroblast cultures of late passages (Figure 3).

In line with our findings, demonstrating the increase in the levels of pAktSer473 and Stat3 in (pre)senescent human dermal and pulmonary fibroblasts, are the most recently obtained evidence of an increased expression and/or activation of Akt and Stat3 both in replicative and stress-induced CS. These observations are summarized in Table 1 and together with our data suggest that the abovementioned changes in Akt and Stat3 are typical for senescent cells of various types.

**Table 1:** Evidence for the involvement of Akt and Stat3 in cellular senescence.

**Cells**	**Type of CS**	**Changes in activity/expression**	**Reference**
**IMR90 human lung fibroblasts**	Replicative CS H_2_O_2_-induced CS	Increased Akt-1 and p-Akt-1 levels in senescent cells	[48]
**Human vascular smooth muscle cells (VSMCs)**	Replicative CS	Increased p-Akt level in senescent cells	[49]
**EJ p53-null human bladder cancer cells**	Replicative CS p53-induced CS	Increased p-Akt (pS473 and pT308) protein level in senescent cells	[46]
**TIG3 human fibroblasts**	Replicative CS IL-6-induced or soluble IL-6Rα- induced CS	Stat3 was constitutively activated in senescent cells (both with or without exogenous IL-6/ IL-6Rα stimulation)	[50]
**Human umbilical vein endothelial cells (HUVECs)**	TNFα-induced CS	Increased p-Stat1 and p-Stat3 levels in senescent cells	[51]
**IPF-derived lung fibroblasts**	Replicative CS	Hyperphosphorylation of Stat3 in IPF-derived lung fibroblasts with features of CS	[52]

Apart from their “canonical” functions, Akt and Stat3 could be linked to CS by other activities. For example, a recent study by Kim et al. (2017) suggests that Akt activation is crucial not only for promoting cell survival but also for induction of SASP [46]. On the other hand, binding of non-phosphorylated Stat3 (but not pStat3!) to regulatory regions of pro-apoptotic genes with subsequent inhibition of their expression, results in an increased resistance to apoptosis [47]. The latter could be also promoted through the c-Met partner BAG1 (BCL2 Associated Athanogene 1), which enhances the anti-apoptotic effects of Bcl2 (GeneCards – Human Gene Database; https://www.genecards.org/). In the model of stress-induced premature CS, we found an increased BAG1 protein level in senescent dermal fibroblasts vs. “young” cultures (data not shown).

In summary, c-Met seems to be mechanistically linked to CS and could serve as a marker of CS. Considering the anti-apoptotic and SASP-related activities of Akt and Stat3, the findings of this study indicate that c-Met could contribute through its downstream targets or partners to at least two major phenotypical features of CS – resistance to apoptosis and senescence-associated secretory phenotype (SASP). The role of c-Met and related proteins in CS appears to be an important point for further investigation.

## MATERIALS AND METHODS

### Cell cultures

Primary cultures of human dermal and pulmonary fibroblasts (obtained from ScienCell, Carlsbad, CA, US) were grown under standard conditions (37 °C, 5% CO_2_) in Dulbecco’s modified Eagles medium (DMEM), supplemented with 10% fetal bovine serum, 1% L-glutamine, and 1% penicillin/streptomycin. All products for cell cultures were from Biological Industries, Beit Haemek, Israel. The cultures were inspected every day under inverted phase-contrast microscope (Primo Vert, Zeiss) and cells were passaged 1:2 upon 75–80% confluence. The number and concentration of viable cells were calculated using Trypan blue exclusion assay. Replicative CS was achieved by serial passaging. The cells were defined as pre-senescent or senescent, based on a dramatic inhibition of cell proliferation or cell growth arrest, respectively; typical CS morphology, and expression of the CS marker SA-β-gal. The SA-β-gal assay was carried out using the Sigma Aldrich SA-β-galactosidase detection kit (GALS), according to the manufacturer’s protocol, with subsequent visualization using the Primo Vert microscope.

### Preparation of cell lysates

Cells were washed with ice-cold PBS and scraped into ice-cold lysis buffer with phosphatase inhibitor cocktail (Stratech Scientific Ltd., CAT# B15001-BIT). Lysates were centrifuged at 14,000 rpm for 10 min at 4 °C, and supernatants were collected. Total protein concentration in each sample was determined using Bradford protein assay (Bio-Rad Laboratories, CAT# 5000006). Quantified samples were then analyzed by Western blotting.

### Western blotting

Equal amounts of protein were separated on 10% SDS-polyacrylamide gels, and electrophoretically transferred into PVDF membranes (Bio-Rad, #1704157). Membranes were blocked for 1 h in 5% BSA in Tris-buffered saline (TBS)-Tween and then incubated with primary antibodies in 5% BSA TBS-Tween. The following primary antibodies were used (all from Cell Signaling Technology): Met (D1C2) Rabbit mAb (#8198), phospho-Met (Tyr1234/1235) Rabbit mAb (#3077), pan-Akt (C67E7) Rabbit mAb (#4691), phospho-Akt (Ser473) (D9E) Rabbit mAb (#4060), and Stat3 (D3Z2G) Rabbit mAb (#12640). Membranes were then incubated with rabbit horseradish peroxidase (HRP)-conjugated secondary antibodies (1:20,000, GE Healthcare) diluted in blocking solution. Protein-antibody complexes were detected by chemiluminescence with ECL supernova (Cyanagen), and images were captured with the Azure C300 Chemiluminescent Western Blot Imaging System, Azure Biosystems.
